# Sicherheitsaspekte bei der Behandlung mit Clozapin: Monitoring und Rechallenge – eine narrative Übersicht

**DOI:** 10.1007/s40211-023-00473-0

**Published:** 2023-06-30

**Authors:** Stefan J. Berger, Alex Hofer

**Affiliations:** Department für Psychiatrie, Psychotherapie, Psychosomatik und Medizinische Psychologie, Univ.-Klinik für Psychiatrie I, Medizinische Universität Innsbruck, Anichstr. 35, 6020 Innsbruck, Österreich

**Keywords:** Clozapin, Monitoring, Rechallenge, Clozapine, Monitoring, Rechallenge

## Abstract

**Hintergrund:**

Auf Grund seiner unvergleichbaren Wirksamkeit bei therapieresistenten schizophrenen Störungen ist der Abbruch einer Behandlung mit Clozapin häufig mit einer erheblichen Verschlechterung der Krankheitssymptomatik, aber auch mit einem erhöhten Suizidrisiko verbunden. Ziel der vorliegenden Übersichtsarbeit ist es, auf Basis aktueller Fachliteratur verschiedene Monitoring-Empfehlungen zusammen zu fassen, um diese Therapie gegebenenfalls trotz auftretender unerwünschter Arzneimittelwirkungen (UAW) fortsetzen zu können. Des Weiteren wird ausgearbeitet, wann eine unterbrochene Therapie mit Clozapin wieder aufgenommen werden kann (Rechallenge) und wann ein definitiver Behandlungsabbruch erfolgen muss.

**Material und Methoden:**

Die Datenbank Medline sowie die Guideline for the use of clozapine 2013 der Netherlands Clozapine Collaboration Group und die S3-Behandlungsleitlinie Schizophrenie der Deutschen Gesellschaft für Psychiatrie und Psychotherapie, Psychosomatik und Nervenheilkunde e.V. wurden nach relevanter Literatur untersucht, die letzte Abfrage erfolgte am 28.04.2023.

**Resultate:**

Bei Entwicklung einer Agranulozytose oder einer Kardiomyopathie muss die Behandlung mit Clozapin beendet werden und sollte auch im weiteren Verlauf nicht wieder aufgenommen werden. Dem gegenüber kann eine auf Grund einer Myokarditis bzw. einer unter der Behandlung auftretenden Verlängerung der QTc-Zeit abgebrochene Behandlung mit Clozapin bei regelrechter linksventrikulärer Funktion bzw. nach Normalisierung der QTc-Zeit gegebenenfalls fortgesetzt werden. Andere UAW stellen in der Regel keine absolute Kontraindikation für eine Rechallenge dar, erfordern jedoch häufig den adjuvanten Einsatz zusätzlicher pharmakologischer und nicht-pharmakologischer Maßnahmen.

**Schlussfolgerung:**

Unter Berücksichtigung verschiedener Monitoring-Empfehlungen kann die Beendigung einer Behandlung mit Clozapin häufig verhindert bzw. eine auf Grund von UAW abgebrochene Behandlung mit Clozapin wieder aufgenommen werden.

## Abstract (en)

**Background:**

Due to its unique efficacy in treatment-resistant schizophrenia, discontinuation of treatment with clozapine is frequently associated with a significant worsening of symptoms, but also with an increased risk of suicide. Based on the literature, this review aims at summarizing different monitoring recommendations in order to be able to continue this therapy despite the occurrence of side effects. In addition, we provide recommendations when rechallenge of a previously stopped treatment with clozapine can be considered and when a definite discontinuation must take place.

**Material and Methods:**

Medline, the Guideline for the use of clozapine 2013 of the Netherlands Clozapine Collaboration Group, and the S3 Guideline for Schizophrenia of the German Association of Psychiatry, Psychotherapy and Psychosomatics were searched for relevant literature, the last query dating from April 28th, 2023.

**Results:**

If agranulocytosis or cardiomyopathy develops, treatment with clozapine must be discontinued and should not be resumed thereafter. In contrast, treatment with clozapine which had to be discontinued due to myocarditis or prolongation of the QTc interval may be continued if left ventricular function is normal or after normalization of the QTc interval. Other side effects are usually not absolute contraindications for rechallenge but often require the adjunctive use of additional pharmacologic and non-pharmacologic measures.

**Conclusion:**

Taking into consideration various monitoring recommendations, cessation of treatment with clozapine can often be prevented or treatment with clozapine that has been discontinued due to side effects can be resumed.

## Einleitung

Auf die Erstsynthese im Jahr 1958 folgte zunächst eine sprunghafte Entwicklung der Behandlung schizophrener Störungen mit Clozapin. Die „finnische Epidemie“ im Jahr 1975 zeigte jedoch, wie gravierend unerwünschte Arzneimittelwirkungen (UAW) unter dieser Behandlung sein können und führte in weiterer Folge zu einer Marktrücknahme. Nach der erneuten Zulassung 1989/1990 in den USA folgten unzählige Studien zu verschiedenen UAW und zu deren Management, da die überlegene Wirksamkeit von Clozapin immer wieder aufs Neue bestätigt wurde [1]. Aufgrund der Tatsache, dass eine vergleichbare Wirksamkeit anderer Antipsychotika bei therapieresistenten schizophrenen Störungen (TRS) nicht konsistent nachgewiesen werden konnte, stellt sich im klinischen Alltag die Frage, wann und ob eine auf Grund von UAW abgebrochene Behandlung mit Clozapin wieder aufgenommen werden kann (Rechallenge).

Wie jede Behandlung folgt die Therapie mit Clozapin immer einer Abwägung von Nutzen und Risiko. Gleiches gilt für einen Therapieabbruch bei Auftreten von UAW, wobei die Abbruchrate besonders im ersten Behandlungsjahr sehr hoch ist (bis zu 65 %) [2] und häufig vermieden werden könnte [3]. Bei beinahe der Hälfte der Fälle zieht ein abruptes Beenden dieser Therapie eine erhebliche Verschlechterung der Krankheitssymptomatik [4] und ein erhöhtes Suizidrisiko nach sich [5], so dass die Behandlung nach Möglichkeit fortgesetzt bzw. wieder aufgenommen werden sollte [2, 5–7]. Basierend auf ausgewählter Fachliteratur widmet sich der zweite Teil dieser Übersichtsarbeit deshalb dem Monitoring bei laufender Therapie bzw. der Wiederaufnahme einer unterbrochenen Behandlung mit Clozapin [8–10].

## Methodik

Erstmalig im März 2022 wurde in Medline die Suchformel (clozapine) AND (monitoring OR rechallenge) AND (agranulocytosis OR thrombocytopenia OR infection OR cardiotoxicity OR cardiomyopathy OR myocarditis OR thromboembolism OR metabolic OR constipation OR seizures OR hypersalivation OR renal) eingegeben. Diese Abfrage ergab über 1000 Einträge, so dass die Suchbegriffe auf Titel und Abstracts eingeschränkt wurden, woraus letztlich 245 Arbeiten resultierten. Diese Suche wurde in weiterer Folge mehrmals wiederholt, zuletzt am 28.04.2023. Zusätzlich wurden spezifische Fragestellungen abgefragt und die Bibliografie der verwendeten Artikel nach weiteren Hinweisen durchsucht. Daneben wurden die im Internet veröffentlichte S3-Behandlungsleitlinie Schizophrenie der Deutschen Gesellschaft für Psychiatrie und Psychotherapie, Psychosomatik und Nervenheilkunde e.V. (DGPPN) und die Guideline for the use of clozapine 2013 der Netherlands Clozapine Collaboration Group hinzugezogen.

## Resultate

### Monitoring

#### Pneumonie-Monitoring

Aufgrund fehlender Fachliteratur beschränken sich die Monitoring-Empfehlungen für die Pneumonie auf die intensive Aufklärung von Patient:innen und deren Angehörigen. Diese sollen auf Infektionszeichen wie Fieber achten und bei deren Auftreten umgehend die behandelnden Ärzt:innen aufsuchen. Diese sind dazu angehalten, besonders auf Entzündungszeichen und Erhöhungen des C‑reaktiven Proteins (CRP) zu achten (pathophysiologische Details sind im ersten Teil dieser Übersichtsarbeit beschrieben). Bei fehlender Möglichkeit einer weiteren Abklärung sollte die Clozapin-Dosis halbiert werden [11].

#### Agranulozytose-Monitoring

Für diese UAW gibt es verpflichtende hämatologische Kontrollen mit definierten Grenzwerten, welche im ersten Teil dieser Übersichtsarbeit bereits genannt wurden. Das Intervall der Blutbildkontrollen unterscheidet sich von Land zu Land, im deutschen Sprachraum sind in den ersten 18 Behandlungswochen wöchentliche Kontrollen und anschließend monatliche Kontrollen vorgeschrieben. Wichtig bei der Beurteilung der Leukozyten-Werte und der absoluten Neutrophilenzahl (ANC) ist jedoch die Unterscheidung zwischen der „Normalbevölkerung“ und Patient:innen mit benigner ethnischer Neutropenie [12].

#### Allgemeines kardiovaskuläres Monitoring

Grundsätzlich sollte bei allen Patient:innen vor Beginn der Behandlung mit Clozapin, aber auch bei Änderungen der Dosis ein Elektrokardiogramm durchgeführt werden. Des Weiteren sollte nach Verabreichung der ersten Dosis über 3–6 h eine Kontrolle von Blutdruck, Puls und Temperatur erfolgen. Bei rascher Titration sollten diese Untersuchungen zweimal täglich durchgeführt werden, bei zurückhaltender Titration ist eine einmalige tägliche Durchführung ausreichend. Anschließend sollten diese Untersuchungen bis zum Erreichen der Erhaltungsdosis zweiwöchentlich erfolgen, danach im Zuge der hämatologischen Kontrollen [12]. Bei unter der Behandlung mit Clozapin auftretender Veränderung der QTc-Zeit ist eine Verlängerung um maximal 60 ms tolerierbar. Das Risiko für eine QTc-Verlängerung > 500 ms (obere Normgrenze bei Männern: 450 ms, bei Frauen: 460 ms) unter einer Monotherapie mit Clozapin ist insgesamt gering und wird vor allem bei Plasmaspiegeln oberhalb des orientierenden therapeutischen Bereiches (> 600 ng/ml) bzw. bei zusätzlicher Verabreichung anderer potenziell QTc-Zeit verlängernder Substanzen beobachtet [13].

#### Myokarditis-Monitoring

Aufgrund der Tatsache, dass 75 % der Clozapin-induzierten Myokarditisfälle in den ersten 4 Behandlungswochen und die überwiegende Mehrheit bis zum Ende des zweiten Behandlungsmonats auftreten, ist ein adäquates Monitoring vor allem zu Beginn der Behandlung wichtig und sollte im Rahmen des wöchentlichen hämatologischen Monitoring-Protokolls erfolgen [14]. Die Kombination einer Erhöhung von CRP (> 100 mg/L) und Troponin (mehr als 2fach) weist in der Diagnostik einer Clozapin-induzierten Myokarditis eine Sensitivität von nahezu 100 % auf [15]. Bei leichten Erhöhungen dieser Werte sollte die Titration unterbrochen werden und eine tägliche Bestimmung von CRP und Troponin erfolgen. Die frühzeitige Diagnostik und nachfolgende Intervention einer Clozapin-induzierten Myokarditis ist mit einem guten Outcome und der grundsätzlichen Möglichkeit einer Rechallenge verbunden [10]. Bei klinischem Verdacht und normalen Laborwerten kann eine kardiale Magnetresonanztomographie indiziert sein, da diese eine besonders hohe diagnostische Genauigkeit besitzt [16]. Bei der Verlaufskontrolle einer Clozapin-induzierten Myokarditis sollte die Echokardiographie zum Einsatz kommen [15].

#### Kardiomyopathie-Monitoring

Entwickelt sich unter Clozapin-Einnahme eine Kardiomyopathie, handelt es sich in der überwiegenden Mehrheit um die dilatative Form (DCM), die häufig asymptomatisch verläuft [17]. Eine routinemäßige Bestimmung des N‑terminalen pro brain natriuretic peptide-Werts (NT-proBNP) kann helfen, kardiale Schäden frühzeitig zu entdecken. Es wurde gezeigt, dass NT-proBNP-Werte von > 31,9 pg/ml mit einer Sensitivität von 91 % und einer Spezifität von 73 % auf eine Verminderung der linksventrikulären Ejektionsfraktion hinweisen [18]. Des Weiteren waren NT-proBNP-Werte > 2247 pmol/L und ein hochsensitiver CRP-Wert > 3,90 mg/L bei Patient:innen mit DCM mit einer erhöhten Gesamtmortalität assoziiert [19].

Des Weiteren sollte eine während der Behandlung mit Clozapin auftretende Sinustachykardie mit Herzfrequenz-senkenden Medikamenten behandelt werden (z. B. Betablocker), da diese als ein prädisponierender Faktor für das Auftreten einer Kardiomyopathie gilt [20].

#### Körpergewicht-Monitoring

In den ersten 3 Monaten der Behandlung mit Clozapin sollte der Body Mass Index (BMI) monatlich, dann jeweils alle 3 Monate bis zum Ende des ersten Behandlungsjahres kontrolliert werden. Aufgrund fehlender Grenzwerte soll eine Ernährungsberatung und die Aufforderung zu ausreichender Bewegung durchgeführt werden, auch eine adjuvante Therapie mit z. B. Metformin kann erwogen und gegebenenfalls im Sinne der Prophylaxe auch zeitgleich eingesetzt werden [21, 22].

#### Glucose- und Lipidmonitoring

Vor Beginn der Behandlung mit Clozapin sollten jedenfalls die Nüchternplasmaglukose, das Nüchterncholesterin, das High Density Lipoprotein und die Triglyceride bestimmt werden. Im Anschluss sollte eine monatliche Kontrolle der Nüchternplasmaglukose bis zum Ende des dritten Behandlungsmonats durchgeführt werden, die übrigen Parameter sollten nach 3 Monaten bzw. anschließend halbjährlich kontrolliert werden. Bis zum Ende des ersten Behandlungsjahres sollte das dreimonatliche Kontrollintervall der Nüchternplasmaglukose beibehalten werden, welches anschließend auf ein halbjährliches geändert werden kann. Sind zwei aufeinanderfolgende Messwerte erhöht (Nüchternplasmaglukose: > 108 mg/dl, postprandiale Plasmaglukose > 141 mg/dl), sollten Fachärzt:innen für Innere Medizin hinzugezogen werden und eine entgegenwirkende Therapie mit z. B. Metformin erwogen werden. Sollte eine Erhöhung des Nüchterncholesterins, des High Density Lipoproteins und der Triglyceride auftreten, sind Ernährungsberater:innen und Hausärzt:innen oder Fachärzt:innen für Innere Medizin hinzuzuziehen [8, 21, 23, 24].

#### Obstipation

Während der Behandlung mit Clozapin sollten Ärzt:innen die Patient:innen bei jedem Kontakt nach der Defäkation befragen und sie dazu auffordern, sich bei Problemen sofort zu melden. Bei Auffälligkeiten sollte eine persönliche Untersuchung erfolgen und die Notwendigkeit weiterer Schritte evaluiert werden. Auch eine präventive Gabe von Laxantien (z. B. Makrogol) kann erwogen werden [23].

### Rechallenge und Therapiestopp

#### Rechallenge nach Pneumonie

Aufgrund der Tatsache, dass Clozapin das einzige Antipsychotikum ist, welches nach einer Pneumonie dosisabhängig mit dem Auftreten einer erneuten Pneumonie verbunden ist, birgt eine Rechallenge ein erhebliches Risiko und sollte ausschließlich bei Patient:innen mit höchstakuter Symptomatik und unter strengsten Überwachungsmaßnahmen durchgeführt werden [25].

#### Rechallenge nach Neutropenie oder Aranulozytose

Bei Patient:innen, welche während einer Behandlung mit Clozapin eine Neutropenie (Neutrophilenzahl > 1500/mm^3^) erlitten haben, kann unter erhöhten Sicherheitsbedingungen eine Rechallenge erfolgen [26], ein entsprechender Monitoring-Vorschlag ist in Tab. 1 dargestellt. Die Zeit bis zu einer erneuten Behandlung, die Geschwindigkeit der Titration und die verabreichte Gesamtdosis haben keinen Einfluss auf den hämatologischen Outcome [26, 27].

**Table 1:** 

Vor Rechallenge	Kurz nach Rechallenge (0–6 Monate)	Langzeit-Überwachung (6 Monate und länger)
„Normale“ Leukozyten-Werte über mindestens 2 Wochen sicherstellen	Zweimal wöchentlich vollständiges Blutbild bestimmen	Wöchentlich vollständiges Blutbild bestimmen
Vollständige Laboruntersuchung (Blut-Harnstoff, Elektrolyte, Lipide, Leberwerte, Blutzucker …)	Andere Parameter wöchentlich bestimmen	Andere Parameter monatlich bestimmen
Lithiumtherapie evaluieren	Stationäre Aufnahme für 2–4 Wochen evaluieren	–
Wöchentliche Überwachung von Temperatur, Puls und Blutdruck	Nach Möglichkeit tägliche Überwachung von Temperatur, Puls und Blutdruck	–
Vorliegen anderer Krankheiten abklären; nach Möglichkeit andere Medikamente absetzen und Einbeziehung von Hämatolog:innen und des Clozapin-Herstellers	Überwachung vorbekannter Erkrankungen; nach Möglichkeit weitere Medikamente vermeiden	Wöchentliche Kontrolle vorbekannter Erkrankungen
Patient:innen über Risiken aufklären	Patient:innen auffordern, sich selbst zu überwachen	Patient:innen auffordern, sich selbst zu überwachen

Bei Patient:innen, welche während einer Behandlung mit Clozapin eine Agranulozytose entwickelt haben, sollte auf Grund des hohen Risikos für das Auftreten einer erneuten Blutdyskrasie kein weiterer Behandlungsversuch mit dieser Substanz erfolgen [26].

#### Rechallenge nach Myokarditis und Kardiomyopathie

Eine routinemäßige Durchführung einer Rechallenge nach Clozapin-induzierter Myokarditis wird trotz erfolgreicher Umsetzung in mehr als 60 % der beschriebenen Fälle [26, 28] nicht generell empfohlen, ist aber grundsätzlich vor allem bei Patient:innen möglich, die keine oder nur eine leichte linksventrikuläre Dysfunktion aufweisen und engmaschig überwacht (Troponin I/T + CRP) werden können. Aufgrund der Tatsache, dass erhöhte CRP-Werte (> 25–50 mg/L) zu einer Down-Regulation hepatischer und extrahepatischer CYPs und somit zu erhöhten Clozapin-Konzentrationen führen, sollte abgewartet werden, bis sich diese normalisieren. Zudem sollte die Titration im stationären Setting und langsam erfolgen [26, 28, 29].

Dem gegenüber ist bei einer Kardiomyopathie, bei der es sich um eine schwere und potenziell letale UAW handelt, eine Rechallenge im Regelfall nicht gerechtfertigt. Sie kann bei Patient:innen mit höchstakuter Symptomatik im Ausnahmefall unter strengsten Überwachungsmaßnahmen erwogen werden, es sind jedoch weitere Studien notwendig [30].

#### Rechallenge nach orthostatischer Hypotension

Bei Auftreten von anhaltenden malignen Synkopen sollte die Behandlung mit Clozapin abgebrochen werden. Nach Ausschluss alternativer Ursachen kann eine Rechallenge erfolgen, allerdings muss auf eine ausreichende Hydrierung und eine adäquate anti-orthostatische Medikation geachtet werden. Zudem sollte die Titration langsam durchgeführt werden und eventuell eine Dosisreduktion erfolgen [3].

#### Rechallenge nach metabolischen UAW

Ein Abbruch der Clozapin-Behandlung bei Auftreten von Gewichtszunahme sollte bei Fehlen anderer UAW im Regelfall nicht erfolgen. Sollte eine Beendigung der Behandlung notwendig sein, kann mit einer medikamentösen und verhaltenstherapeutisch unterstützten Gewichtskontrolle eine Rechallenge mit Clozapin erfolgen [3]. Analog dazu gilt ein Diabetes mellitus während der Behandlung mit Clozapin grundsätzlich als beherrschbar [31]. Die Clozapin-Behandlung sollte ab Nüchternplasmaglukose-Werten > 350 mg/dl abgebrochen werden, jedoch kann nach medikamentöser Einstellung des Diabetes eine Rechallenge erfolgen [3, 31, 32].

Ein Abbruch der Clozapin-Behandlung aufgrund des Auftretens einer Dyslipidämie sollte bei Fehlen anderer UAW im Regelfall ebenfalls nicht erfolgen. Sollte ein Abbruch notwendig sein, kann in Kombination mit einer medikamentösen Lipidtherapie und ausreichender Bewegung eine Rechallenge mit Clozapin erfolgen [3, 32].

#### Rechallenge nach hepatischer Funktionsstörung/gastrointestinaler Hypomobilität

Bei Auftreten von 2–3fach über der oberen Normgrenze liegenden Transaminase-Werten sollte die Clozapin-Behandlung zunächst abgebrochen werden. In weiterer Folge ist nach Normalisierung der Leberenzym-Werte eine Wiederaufnahme dieser Behandlung möglich [3].

Auch bei Auftreten eines Ileus bzw. Subileus sollte Clozapin bis zu dessen erfolgreicher Behandlung abgesetzt werden. Bei Wiederaufnahme der Behandlung mit Clozapin sollte eine Ernährungsberatung erfolgen sowie der Einsatz von Laxantien erwogen werden [3, 26].

#### Rechallenge nach Krampfanfällen

Ein Abbruch der Clozapin-Behandlung bei Auftreten von Krampfanfällen sollte bei Fehlen anderer UAW im Regelfall nicht erfolgen. Sollte ein Abbruch notwendig sein, kann nach einer Verringerung der Dosis und eventuell unter adjuvanter Verordnung von Antiepileptika ohne Auswirkungen auf die myelopoetischen Zellen eine Rechallenge erfolgen [3].

#### Rechallenge nach malignem neuroleptischen Syndrom (MNS)

Bei Auftreten des potenziell tödlichen MNS muss die Behandlung mit Clozapin abgebrochen werden. Es gibt durchwegs positive Zahlen zur Rechallenge mit Clozapin, jedoch sollte ein medianer Sicherheitsabstand von 90 Tagen vom Abbruch bis zum Wiederbeginn der Behandlung eingehalten werden, während dessen alternative Antipsychotika eingesetzt werden sollen. Neben einer ausreichenden Hydrierung sollten Patient:innen auf Anzeichen einer sich entwickelnden Hyperthermie, Rigidität, anderer extrapyramidal-motorischer Störungen, Dysphagie, Dysarthrie, autonomer Störungen oder Anzeichen mentaler Veränderungen untersucht werden. Neben der langsamen Titration könnte auch eine serielle Bestimmung der Kreatinkinase hilfreich sein, um eine erfolgreiche Rechallenge zu gewährleisten [3, 26].

## Conclusio

Auf Grund seiner nachgewiesenen einzigartigen Wirksamkeit bei TRS kann die Beendigung einer Behandlung mit Clozapin den Patient:innenoutcome maßgeblich beeinträchtigen. Es ist deshalb nicht erstaunlich, dass Clozapin gemeinsam mit Olanzapin die zweitniedrigste Behandlungsabbruchrate aufweist [33]. Wird die Entscheidung für einen Therapieabbruch von Seiten der behandelnden Ärzt:innen getroffen, werden in knapp einem Viertel der Fälle medizinische Gründe, wie zum Beispiel UAW, angeführt [5]. Diese stellen jedoch häufig keinen zwingenden Grund für einen Behandlungsabbruch dar [3], so dass in dieser Arbeit auf Basis aktueller Fachliteratur zunächst verschiedene Monitoring-Empfehlungen zusammengefasst wurden, um die Therapie gegebenenfalls trotz auftretender UAW fortsetzen zu können. Bei Agranulozytose oder Kardiomyopathie muss Clozapin jedenfalls sofort abgesetzt werden und sollte auch im weiteren Verlauf nicht wieder zum Einsatz kommen. Bei einer im Zusammenhang mit Clozapin auftretenden Myokarditis bzw. einer unter der Behandlung auftretenden Verlängerung der QTc-Zeit > 60 ms oder einer QTc-Zeit > 500 ms muss die Behandlung zunächst ebenfalls beendet werden, kann in weiterer Folge jedoch bei regelrechter linksventrikulärer Funktion bzw. nach Normalisierung der QTc-Zeit wieder aufgenommen werden. Andere UAW stellen in der Regel keine absolute Kontraindikation für die Wiederaufnahme einer vorbestandenen, abgebrochenen Therapie mit Clozapin dar, so dass diese bei klinischer Notwendigkeit und unter Einsatz adjuvanter pharmakologischer und nicht-pharmakologischer Maßnahmen erfolgen kann.
